# Predicting gene-specific regulation with transcriptomic and epigenetic single-cell data

**DOI:** 10.1093/bioinformatics/btag299

**Published:** 2026-05-15

**Authors:** Laura Rumpf, Fatemeh Behjati Ardakani, Dennis Hecker, Marcel H Schulz

**Affiliations:** Institute for Computational Genomic Medicine, Goethe University Frankfurt, Frankfurt am Main, Hesse 60590, Germany; Institute for Computational Genomic Medicine, Goethe University Frankfurt, Frankfurt am Main, Hesse 60590, Germany; Institute for Computational Genomic Medicine, Goethe University Frankfurt, Frankfurt am Main, Hesse 60590, Germany

## Abstract

**Motivation:**

Analysis of single cell ATAC-seq and RNA-seq data has allowed to gain unprecedented insights into gene regulation by allowing to define cell type-specific regulatory regions and their effects on gene expression. While powerful, such analysis is challenging due to the inherent sparsity of single cell data.

**Results:**

We present a new approach, MetaFR, to learn gene-specific models that link open-chromatin variation from scATAC-seq data to gene expression from scRNA-seq. Using efficient regression trees, we illustrate that accurate expression prediction models can be learned on the single-cell or meta-cell level. Validation was done using fine-mapped eQTLs. Meta-cell models were found to outperform single-cell models for most genes. Comparison to the SOTA method SCARlink revealed advantages of MetaFR in terms of runtime and prediction performance. MetaFR thus allows time-efficient analysis and obtains reliable models of gene expression prediction, which can be used to study gene regulation in any organism for which scRNA-seq and scATAC-seq data is available.

**Availability and implementation:**

MetaFR is available under https://github.com/SchulzLab/MetaFR.

## Introduction

A prevailing challenge in the field of epigenetics stems from the limited understanding of transcriptional regulation diversity. Cis-regulatory elements (CREs) such as enhancers or silencers play a central role in gene regulatory programs that shape cellular development and identity (Vaquerizas *et al.* 2009, Yao *et al.* 2015, Kim and Wysocka 2023, Preissl *et al.* 2023). They have been linked to several diseases and have been shown to be promising candidates for molecular targets in therapeutic interventions (Yao *et al.* 2015, Sgro and Blancafort 2020, Bacabac and Xu 2023).

Genome-wide measurements of transcriptional and epigenetic activity have led to the development of numerous approaches for linking enhancers to genes and predicting gene expression. The integrative analysis of both modalities measured in individual cells enables cell type-specific insights into gene regulation mechanisms (Stuart *et al.* 2019). Epigenome-expression modeling approaches can be divided into two categories: gene-agnostic and gene-specific (Scherer *et al.* 2021). Gene-agnostic approaches model all genes jointly, regarding them as a uniform set of training instances. For instance, sequence-based approaches utilize the DNA sequence from a single biological sample as input to predict epigenetic signals, gene expression levels, or both, genome-wide (Zhou and Troyanskaya 2015, Kelley *et al.* 2018, Agarwal and Shendure 2020, Hingerl *et al.* 2025). By doing so, these approaches bypass the need for large numbers of biological samples, as they exploit the complete genetic information of the genome. However, they do not explicitly resolve gene-specific regulatory architecture, such as the precise locations of CREs and their contribution to gene expression.

In contrast, gene-specific approaches leverage the variability of the epigenetic signal across samples, usually within a window surrounding a gene, to predict gene expression. In this case, one model per gene is learned to associate the epigenome with the gene expression (Hait *et al.* 2018, Ma *et al.* 2020, Granja *et al.* 2021, Schmidt *et al.* 2021, Chen and Keleş 2024, Mitra *et al.* 2024, Shinkai *et al.* 2025). While gene-specific approaches are computationally intensive due to the large number of models that need to be trained, they are particularly well suited to harness the statistical power provided by the large number of samples in single-cell data. Accordingly, several gene-specific methods have been developed for single-cell data.

In general, these approaches can be divided into univariate and multivariate (Chen and Keleş 2024). The univariate or correlation-based approaches model pairwise enhancer-gene associations (Ma *et al.* 2020, Granja *et al.* 2021, Sakaue *et al.* 2024, Su *et al.* 2025), while the multi-variate methods regress over all regions within a window around a gene (Chen and Keleş 2024, Mitra *et al.* 2024). The advantage of multivariate over correlation-based methods lies in their ability to capture co-regulatory effects, that is, they can take into account the combined influence of several regulatory elements on gene expression rather than considering each element in isolation. This is particularly relevant in single-cell data, where the large number of cells enables modeling of cell type-specific regulation and helps disentangle the joint effects of multiple regulatory regions.

The state-of-the-art multivariate method SCARlink (Mitra *et al.* 2024) utilizes multiome RNA-seq and ATAC-seq data to predict gene expression with regularized Poisson regression. One limitation of this method is its restriction to positive coefficients for easier model learning and interpretation. Consequently, SCARlink is not able to capture suppressive interactions, which are essential for transcriptional regulation (Courey and Jia 2001, Payankaulam *et al.* 2010). Chen and Keleş 2024 benchmarked SCARlink against other multivariate approaches for identifying enhancer-gene associations, showing that although SCARlink performed best, no method fully captures how chromatin accessibility regulates gene expression.

A particular challenge is the inherent sparsity of single-cell data. While a standard scRNA-seq matrix contains 55−95% zeros, scATAC-seq data is even more sparse with 90−99% zeros (Chen *et al.* 2019). As a result, extracting biologically meaningful insights remains challenging. A common strategy to address this problem is to aggregate the cells into meta-cells according to the similarity of their gene activity profiles (Baran *et al.* 2019, Ben-Kiki *et al.* 2022, Bilous *et al.* 2022, Persad *et al.* 2023). Persad *et al.* (2023) evaluated the effect of meta-cell aggregation using a correlation-based approach. However, for multivariate models, the impact of meta-cell aggregation on gene expression prediction accuracy remains unexplored. An alternative strategy to reduce sparsity is imputation, which estimates missing values at the single-cell level for RNA and ATAC (van Dijk *et al.* 2018, Li *et al.* 2021, Raevskiy *et al.* 2023). While imputation methods can recover signals, their performance can vary across sequencing protocols and data characteristics (Hou *et al.* 2020). In contrast, meta-cell aggregation provides a simple and technology-agnostic representation.

We developed a fully automated Nextflow pipeline, **MetaFR** (Meta-Cell Forest Regression), that leverages single-cell RNA-seq and ATAC-seq data to build gene-specific regression trees for gene expression prediction. These trees avoid overfitting and are fast, and we show that they outperform the state-of-the-art method SCARlink (Mitra *et al.* 2024) in both accuracy and runtime. Further, we assessed the impact of meta-cell creation on model performance. Applying a signal aggregation strategy significantly improved the prediction accuracy and the ability to find biologically meaningful interactions. Finally, we evaluated gene characteristics that have an impact on performance differences between meta-cell and single-cell models, such as number of TSSs or gene expression sparsity. Our pipeline is available for the community via the MetaFR repository: https://github.com/SchulzLab/MetaFR.

## Results

### Modeling gene-specific expression with MetaFR

**Figure 1:**
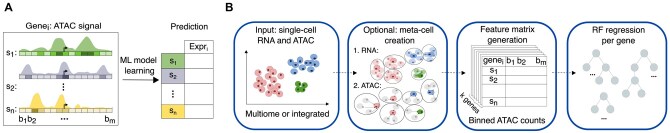
Overview of the MetaFR pipeline. (A) General learning setup: gene-specific models are learned using the epigenetic signal across samples $s_{1}\ldots s_{n}$ in a window around a gene. The epigenetic signal is quantified in equal-sized bins $b_{1}\ldots b_{m}$ and used to predict expression of gene${\;}_{i}$ ($\text{Exp}r_{i}$). (B) MetaFR is an automated Nextflow pipeline that utilizes scRNA and scATAC data to predict gene expression in a gene-specific manner on single-cell or meta-cell level using Random Forest (RF)-regression trees. For the single-cell setup, multiome data is required. When the meta-cell creation step is applied, unpaired epigenetic and transcriptomic data can be leveraged. In this case, RNA and ATAC cells have to be integrated beforehand to be aligned in a shared feature space.

We developed a fully automated Nextflow pipeline, MetaFR, to accurately predict gene expression from single-cell transcriptomic and epigenomic data (Fig. 1). Supplementary Figure 1 provides a detailed flow chart. Each gene is assigned its own model, trained in a gene-specific manner. The features of the gene expression prediction models are defined as equal-sized non-overlapping bins $b_{1}\ldots b_{m}$ that hold the quantified epigenetic signal for each sample $s_{1}\ldots s_{n}$ in a window around a gene (Fig. 1A). Within this study, we use a 1 MB window surrounding the most $5^{\prime }$ GENCODE (The GENCODE consortium 2019) TSS of a gene, and examine varying bin sizes of 100 bp, 250 bp, and 500 bp. A 1 MB genomic window was used, following previous studies showing that this range effectively captures distal cis-regulatory interactions while remaining computationally feasible (Sakellaropoulos *et al.* 2024, Jeong and Bulyk 2025). To predict gene expression we chose a Random-Forest (RF) regression approach, given its ability to model non-linear dependencies and to avoid overfitting, while remaining computationally efficient. The models can be trained on single-cells when multiome data is provided. To address the inherent sparsity of single-cell data, the pipeline allows for cells to be grouped into meta-cells based on their gene expression similarities. If relevant metadata is available, meta-cell clustering can be performed separately within each annotated group to avoid oversmoothing of rare populations. After generating meta-cells, we assign cells from the scATAC-seq dataset to these RNA meta-cells, resulting in final meta-cells that incorporate both modalities. This enables our method to handle unpaired single-cell data (see “Methods” section), where transcriptomic and epigenomic profiles originate from different cells, provided they have been integrated into a shared feature space beforehand. To ensure accurate aggregation, we verified that RNA and ATAC cells from the used 10× multiome PBMC dataset (https://www.10xgenomics.com/datasets/10-k-human-pbm-cs-multiome-v-1-0-chromium-x-1-standard-2-0-0) and multiome brain dataset (Anderson *et al.* 2023) are well aligned in the integrated space (Supplementary Figs. 2 and 4A). For model training, the feature matrix is generated for each gene and contains binned accessibility signal within the window around the gene, either at the single-cell or meta-cell level. For each gene, the trained RF model for expression prediction and a performance report are provided as output. The report contains the training and test error (Mean Squared Error, MSE) as well as the Pearson and Spearman correlation between predicted and actual expression (Fig. 1B). Although all analyses were conducted on paired datasets, pairing information was not used at any step of the meta-cell pipeline or during integration, effectively treating the data as unpaired. To compare the unpaired setting with one that incorporates cell-pair information, we generated meta-cells by clustering cells solely based on their gene expression profiles. Most genes showed comparable performance, a subset showed differences, with neither approach outperforming the other (Supplementary Fig. 3).

### Prediction accuracy increases after meta-cell aggregation

**Figure 2:**
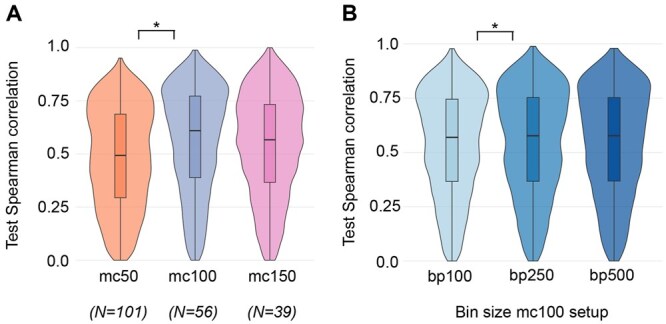
Model performance assessment for meta-cell (mc) MetaFR setups. To assess model accuracy the Spearman correlation was calculated between predicted and actual gene expression on a test set (20% of all data). (A) Correlation on test data for the meta-cell setups with 50, 100 and 150 cells aggregated into one meta-cell for 10 754 genes, where all setups could obtain a model with test correlation >0. (B) Correlation on test data for the meta-cell setup with 100 cells per meta-cell and bin sizes varying between 100, 250 and 500 base-pairs. Center line is median, boxlimits correspond to IQR, whiskers to 1.5× IQR. Outliers are not shown. A paired Wilcoxon signed-rank test was performed between gene sets of different setups (*P*-value ≤ .05 indicated with an asterisk).

We applied our pipeline to a publicly available multiome PBMC dataset from 10× (https://www.10xgenomics.com/datasets/10-k-human-pbm-cs-multiome-v-1-0-chromium-x-1-standard-2-0-0) with approximately 10 000 cells, which is widely used for benchmarking due to its high quality. To assess the generalizability of our approach, we additionally evaluated performance on an independent multiome brain dataset from Anderson *et al.* (2023), comprising 105 332 nuclei isolated from cortical tissue of Alzheimer’s disease and control donors (Supplementary Fig. 4A). To assess the model accuracy in an unbiased manner, we randomly held out 20% of the dataset for model testing. The number of input genes for the training phase was 36 601. We learned for around 19 300 genes for all tested MetaFR setups. The number of models varies slightly between the methods. Figure 2 shows the prediction performance of different MetaFR setups, evaluated by the Spearman correlation between predicted and observed gene expression on the test set. When investigating the impact of grouping different numbers of cells per meta-cell, we found that increasing the meta-cell size leads to an improved prediction accuracy (Fig. 2A, Supplementary Fig. 4B). However, with 150 cells per meta-cell, performance decreases slightly compared to the setting with 100 cells per meta-cell. The decrease can be explained by the number of training instances available after aggregation, as merging more cells into one meta-cell lowers the total number of samples. More precisely, for cell types with medium abundance (150–500 cells), using 150 cells per meta-cell frequently results in all cells being aggregated into a single meta-cell (Supplementary Fig. 5). Since the setup with 100 cells per meta-cell yielded the best performance it was used in all subsequent analyses. A pairwise comparison of the meta-cell setups based on MSE is provided in Supplementary Fig. 6A. Figure 2B depicts model performance for genomic feature sizes of 100, 250, and 500 base-pairs for the best performing meta-cell setup. The results for the single-cell setup can be found in Supplementary Fig. 6B. Overall, the effect of bin size on performance was minor, although larger bins slightly improved the prediction accuracy. In contrast, the runtime per gene in seconds decreased substantially, from approx. 4.2 for 100 bp bins to approx. 3 for 500 bp bins at single-cell level, corresponding to a reduction in total runtime from approximately 42.7 hours to 30.5 hours. Consequently, we chose a feature size of 500 bp for all subsequent analyses. No significant differences in performance were observed across different bin sizes in the brain dataset (Supplementary Fig. 4C). Evaluation of the robustness of model performance to hyperparameter selection (Supplementary Methods) for models trained with 100 cells per meta-cell showed that performance was largely insensitive to hyperparameter choice. The difference in MSE between models with and without hyperparameter-tuning was negligible, indicating that the default configuration already achieves near-optimal performance (Supplementary Fig. 7).

**Figure 3:**
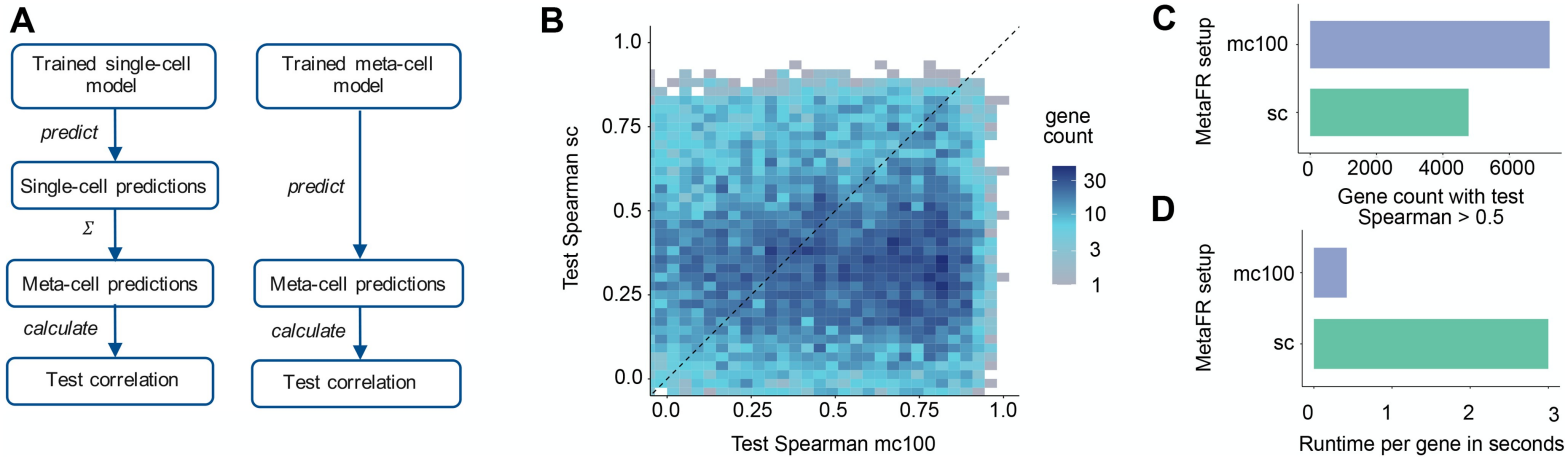
Model performance comparison between single-cell (sc) and meta-cell (mc) MetaFR setups. To assess model accuracy the Spearman correlation was calculated between predicted and actual gene expression on a 20% test set. (A) Workflow for direct comparison of meta-cell and single-cell accuracy: single-cell predictions are aggregated across test cells assigned to the same meta-cell as in the meta-cell setup. (B) Test correlation for meta-cell setup with 100 cells per meta-cell and aggregated single-cell predictions according to (A). (C) Number of genes where a model could be generated with test correlation >0.5. (D) Runtime per gene on an Intel Xeon CPU node with 64 Cores at 2.6–3.3 GHz Frequency and 384 GiB RAM using 25 cores.

In order to evaluate the impact of meta-cell generation on the prediction performance, a comparison was made between aggregated single-cell predictions and meta-cell predictions (Fig. 3). To enable direct comparison between meta-cell and single-cell prediction methods, single-cell predictions were summarized into meta-cell representations. More specifically, the single-cell predictions for cells that belong to the same meta-cell were summed. The cell-to-meta-cell assignment was derived from the meta-cell setup. Finally, the Spearman correlation was calculated between predicted and actual gene expression (Fig. 3A). Overall, one can observe that for the majority of genes the meta-cell predictions achieve higher accuracy than the single-cell predictions, although there is a substantial number of genes for which this is not the case (Fig. 3B). In the brain dataset, meta-cell predictions outperformed single-cell predictions, assessed on the 500 most variable genes (Supplementary Fig. 4D). For the two setups described above, the number of gene models with a test correlation >0.5 was 7217 for the meta-cell setup and 4774 for the single-cell setup (using correlation estimates after aggregation; Fig. 3C). Additionally, the runtime per gene was approximately 0.42 seconds for the meta-cell setup compared to approximately 3 seconds for the single-cell setup (Fig. 3D). This resulted in a total runtime of approximately 4.3 hours for the meta-cell setup compared to 30.5 hours for the single-cell setup.

**Figure 4:**
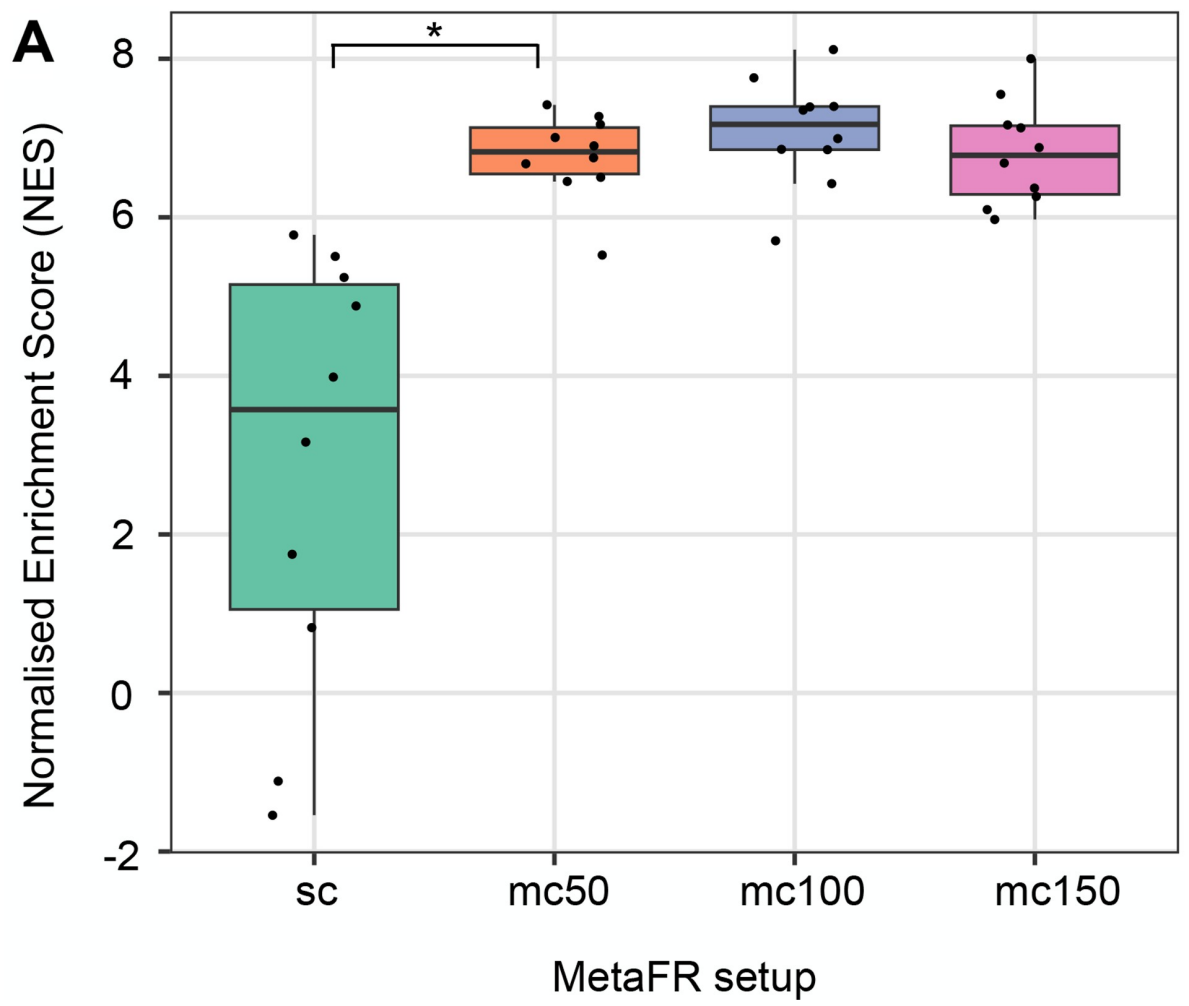
Comparison between single-cell (sc) and meta-cell (mc) MetaFR setups in ability to identify biologically meaningful interactions. (A) Cell type-specific GSEA Normalized Enrichment Score (NES) (Fang *et al.* 2023) of GTEx (The GTEx Consortium 2020) whole blood eQTLs in the top 100 000 ranked interactions of the 1000 most variable genes. Interactions were ranked across genes using IQR-scaled absolute SHAP values. Additionally, a distance decay weighting was applied (Chen and Keleş 2024), since eQTLs are biased to be in close proximity to a gene’s promoter (Mostafavi *et al.* 2023, Arthur *et al.* 2025). Cell types with fewer than 100 cells and fewer than 800 eQTL hits among the most variable genes were excluded. The Center line denotes the median, boxlimits correspond to IQR, whiskers to 1.5× IQR. A paired Wilcoxon signed-rank test was performed between gene sets of different setups. *P*-value ≤ .05 indicated with an asterisk. Supplementary Fig. 8 shows the NES without distance decay weighting.

We evaluated the differences in the ability between the single-cell and meta-cell setups to identify biologically meaningful region-gene interactions (Fig. 4, Supplementary Fig. 8). As ground truth interactions, expression-quantitative trait loci (eQTL) gene interactions were obtained from GTEx (The GTEx Consortium 2020) for the whole blood tissue. To rank the region-gene interactions obtained from the gene models the feature importance was estimated using absolute SHAPley values (Lundberg *et al.* 2020) in a cell type-specific manner (see “Methods” section). We found that, overall, the meta-cell setups achieved significantly higher enrichment than the single-cell setup.

### Gene characteristics explain performance differences between single-cell and meta-cell models

**Figure 5:**
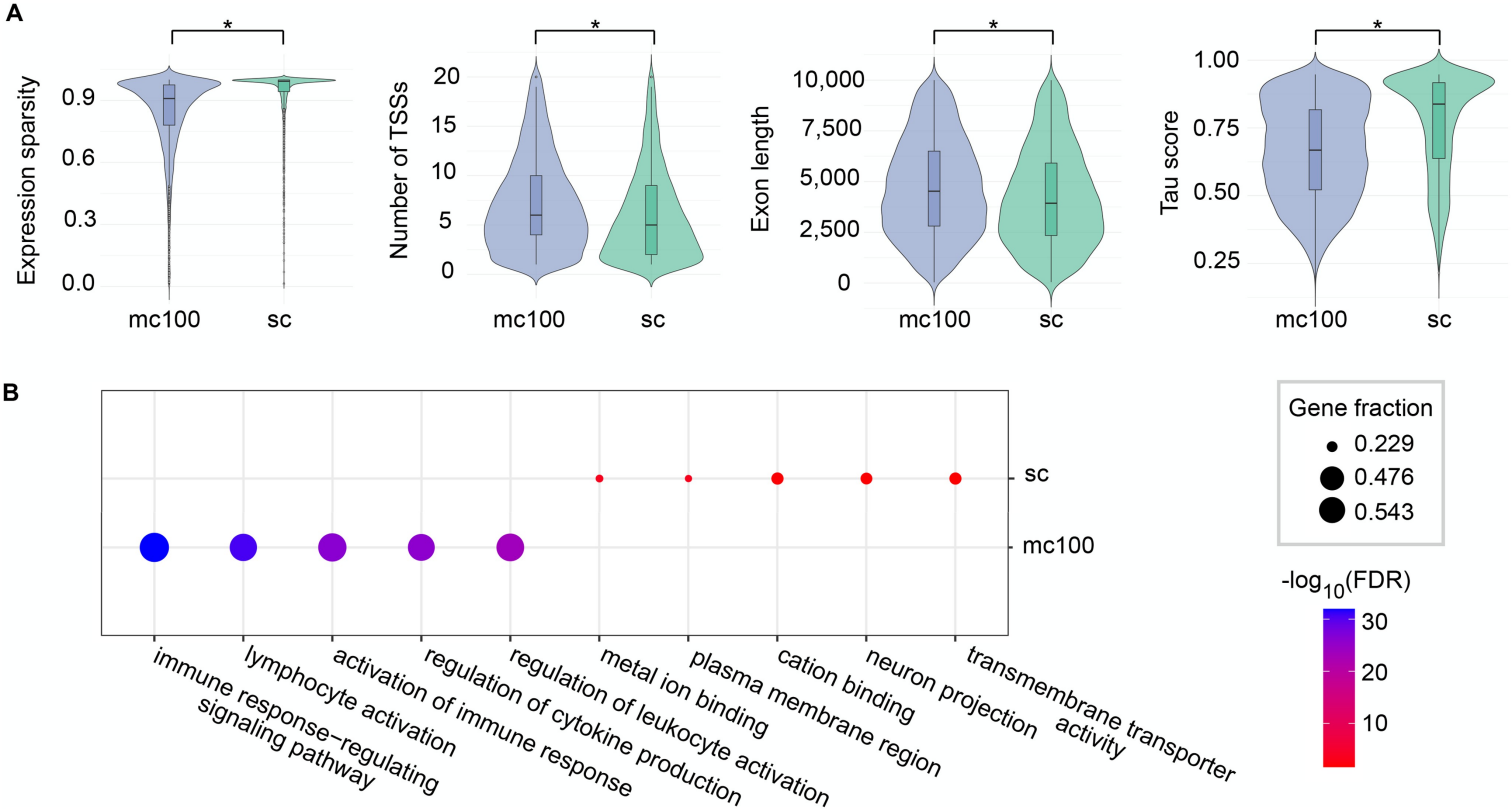
Investigation of gene characteristics that impact performance differences between single-cell (sc) and meta-cell (mc) setups. The best-performing gene sets for the single-cell (4615 genes) and meta-cell setup with 100 cells per meta-cell (7172 genes) are defined as the models that achieve a minimum performance of 0.3 test correlation and where one method outperforms the other by >0.1. (A) Assessed gene characteristics include gene expression sparsity, corresponding to fraction of 0 in the expression in test cells, number of annotated GENCODE (The GENCODE consortium 2019) TSSs, total exon length and cell type specificity. For cell type specificity the Tau score (Yanai *et al.* 2005) was used: $\tau =\frac{\sum_{i=1}^{N}(1-x_{i})}{N-1}$, where *N* is the number of cell types and $x_{i}$ is the gene’s expression in cell type *i* normalized to the maximum expression across all cell types. (Center line is median, box limits correspond to IQR, whiskers to 1.5× IQR. An unpaired Mann-Whitney *U* Test was performed between the best-performing gene sets. *P*-value ≤ .05 indicated with an asterisk). (B) GO enrichment with g: Profiler (Kolberg *et al.* 2023) of best-performing gene-sets. The background was set to the initial gene set of 36 601 genes. High-level terms (depth ≤7) were filtered with R Package ontologyIndex (Greene *et al.* 2017). Displayed GO terms were selected from the top 100 significant terms. All terms can be found in Supplementary Material.

We evaluated which gene characteristics contribute to the differences in prediction accuracy between single-cell and meta-cell MetaFR (100 cells per meta-cell) (Fig. 5). Genes included in the analysis were those with aggregated single-cell (see “Methods” section) or meta-cell test Spearman correlations ≥0.3, and where one setup outperformed the other by at least 0.1 correlation. Figure 5A indicates that single-cell MetaFR performs better for highly sparse genes. In addition, when assessing cell type specificity using the Tau score (Yanai *et al.* 2005), single-cell MetaFR shows improved predictions for genes with higher cell type specificity. Analysis of the number of annotated GENCODE (The GENCODE consortium 2019) TSSs shows that the single-cell setup outperforms meta-cell MetaFR for genes with fewer TSSs and shorter exon lengths. Furthermore, single-cell models tended to perform better for genes with fewer annotated GENCODE transcripts, shorter gene length, and lower number of genes within a 1 MB window (Supplementary Fig. 9A). Genes were stratified into performance groups according to the test correlation, revealing consistent trends across meta-cell and single-cell setups, except that longer genes and denser loci improved meta-cell performance but reduced single-cell performance (Supplementary Fig. 9B and C).

A GO enrichment analysis of the genes that performed best in the single-cell setup identified a total of 15 significant terms. A representative selection of these terms is shown in Fig. 5B. The enriched terms predominantly relate to basic cellular processes, reflecting the core functions captured by the single-cell models. In contrast, 8 804 GO terms were enriched among the best-performing meta-cell genes. Notably, within the top 100 most significant biological process terms, several were cell-type-specific, including processes such as activation of the immune response.

### MetaFR outperforms the state-of-the art method SCARlink in accuracy and runtime

**Figure 6:**
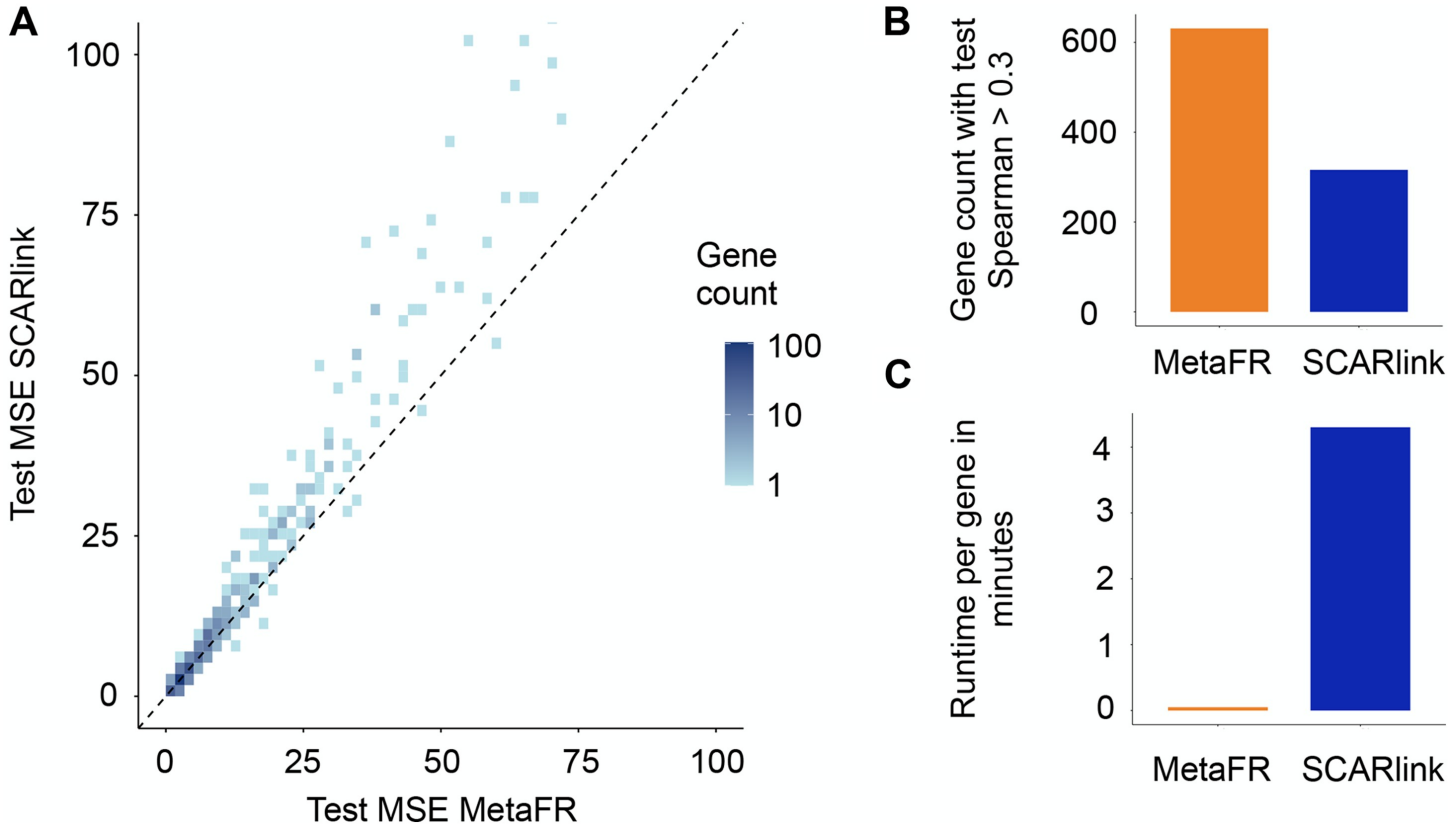
Comparison to state-of-the-art method SCARlink. (A) Test error (MSE) of MetaFR and SCARlink for 495 genes, for which both methods produced a model. (B) Number of genes where a model could be generated with test correlation (Spearman) >0.3 from their respective initial gene sets (see “Methods” section). (C) Runtime per gene on an Intel Xeon CPU node with 64 Cores at 2.6–3.3 GHz Frequency and 384 GiB RAM using 25 cores. The test error and correlation are calculated between predicted and actual gene expression.

We compared MetaFR to the state-of-the-art multi-variate regression approach SCARlink (Mitra *et al.* 2024) regarding the prediction accuracy at single-cell level. The feature setup is identical for both approaches: The epigenetic signal is binned with 500 bp in a window around the target gene. For SCARlink the size of the genomic window varies between genes, since a 250 kb extension upstream and downstream of the gene body is applied. It uses $L_{1}$-regularized Poisson regression as a prediction method. All subsequent analyses are conducted at the single-cell level. To assess the prediction accuracy we calculated the MSE and Spearman correlation between predicted and actual gene expression on the same test cells. Following the default practice for SCARlink, the input gene set was defined as the 2000 most variable genes. A model could be generated for 536 genes. The number of genes where both approaches obtained a model was 495. Regarding the MSE, MetaFR outperforms SCARlink on 354 of those genes (Fig. 6A). The number of genes where a method was able to generate a model with test correlation above 0.3 amounts to 631 for MetaFR and 316 for SCARlink, respectively (Fig. 6B). To control for differences in feature number, MetaFR was retrained with its feature count reduced to the median of SCARlink (Supplementary Fig. 10). Performance differences were similar to those observed using the full feature set, with MetaFR outperforming SCARlink on 334 genes. SCARlink required approximately 4.3 minutes to learn one gene, whereas MetaFR required approximately 3 seconds. Consequently, the total runtime for the respective gene sets was approximately 6 days (143 hours) for SCARlink and 1.3 days (30.5 hours) for MetaFR. For the brain dataset, models were trained for the 500 most variable genes. Overall, prediction performance of the two methods was relatively similar (Supplementary Fig. 4E). Among the 125 genes for which both methods produced a model, MetaFR achieved lower test error more frequently than SCARlink. The runtime per gene for SCARlink was approximately 20.82 minutes, for MetaFR approximately 1.47 minutes. Total runtime for processing 500 genes amounted to approximately 7.23 days (173.5 hours) for SCARlink and 0.5 days (12.15 hours) for MetaFR.

### Transcription factor enrichment reveals cell type-specific regulation

To assess whether the predicted region–gene interactions are enriched for cell type-specific transcription factor (TF) binding, we performed TF enrichment analysis on regions identified with SHAP (Supplementary Methods) using PASTAA (Roider *et al.* 2009). Among the top significant transcription factors, several are consistent with known cell type-specific regulatory programs. Focusing on the most abundant cell type in the dataset, monocytes showed enrichment of EGR1 and SP1, which are involved in monocytic differentiation and CD14 expression, respectively (Zhang *et al.* 1994, Kubosaki *et al.* 2009). In T cells, NFATC2 was significantly enriched, particularly in CD8 TEM-2 cells, and was selected for further locus-level analysis due to its established role in T cell activation (Rengarajan *et al.* 2002). Integrative Genomics Viewer (IGV) visualization (Robinson *et al.* 2011, https://github.com/gladkia/igvShiny) revealed differences in SHAP importance profiles between naive and activated T cells, with comparable absolute importance but opposite directional effects (Supplementary Fig. 11). All significantly enriched TFs for cell types with more than one meta-cell are provided in the Supplementary Material.

## Discussion

We developed MetaFR, a fully automated Nextflow pipeline that generates gene-specific regression-based Random Forest models to predict gene expression from single-cell RNA-seq and ATAC-seq data. Our pipeline allows time-efficient analysis and obtains reliable models of gene expression, which can be used to study gene regulatory elements in any organism for which single-cell RNA-seq and ATAC-seq data is available. We were able to outperform the state-of-the-art gene-specific multivariate method for gene expression prediction SCARlink (Mitra *et al.* 2024) in both prediction accuracy and runtime. We systematically evaluated the impact of meta-cell creation on expression prediction accuracy. Our analysis revealed that meta-cell aggregation leads to a significant increase in model accuracy. Although increasing the number of cells per meta-cell generally improves the performance, it is essential to retain a sufficient number of training samples. Overall, the choice of meta-cell size is highly dataset-specific. It depends on factors such as the number of cells per cell type or experimental conditions. Consistent results across model performance analyses were obtained on an independent multiome brain dataset (Anderson *et al.* 2023), supporting the robustness of the observed trends beyond the 10× PBMC dataset analyzed in this study.

A particular challenge is the presence of rare cell types, which are often aggregated into a single meta-cell, limiting their contribution to model training. In addition, meta-cell generation is strongly influenced by the quality of the integration as ATAC cells are assigned to the closest RNA meta-cell in the integrated space. To ensure the generation of high-quality meta-cells, we evaluated the integration, confirming that RNA and ATAC cells were well aligned. To mitigate the impact of the integration, metadata such as cell type can be incorporated in the clustering process to preserve biological heterogeneity and reduce oversmoothing. Despite the availability of paired measurements in multiome data, explicit pairing does not necessarily reflect the true cell state, given the dynamic and sequential nature of gene regulation (Ma *et al.* 2020, Li *et al.* 2023). Accordingly, incorporating pairing information in the meta-cell approach did not significantly improve performance. However, the single-cell setup relies on paired data, and extending this setting to fully unpaired datasets would require integration methods that establish cell–cell correspondences across modalities.

An advantage of using Random Forest models for prediction of gene expression is their computational efficiency, which facilitates the exploration of different parameter settings. This allows exploration of key modeling choices such as the number of cells per meta-cell, while extensive tuning of Random Forest hyperparameters appears less critical, given the near-optimal performance of the default configuration.

Although overall performance improved with meta-cell aggregation, a substantial subset of genes was better predicted by single-cell models. These genes were lowly expressed, with a median sparsity of around 95%. A possible explanation is that, for highly sparse genes, meta-cell summarization overly reduces expression variability, thereby limiting the model’s ability to learn informative patterns. We found that single-cell models perform better for genes with simpler structure (shorter exon lengths and fewer TSSs). Correlation analysis indicated a negative association between the expression sparsity and exon lengths (≈−0.39), as well as expression sparsity and number of TSS (≈−0.44). Since there are fewer regions to sample during sequencing of shorter exons, sparsity naturally increases. Similarly, genes with higher cell type specificity tend to exhibit increased sparsity.

To further assess differences in the regulatory relevance of predictions from the single-cell and meta-cell setups, we evaluated enrichment with GTEx eQTLs. While GTEx whole-blood eQTLs provide a useful reference for validation, they may miss regulatory effects that are cell type-specific or context-dependent (Kang *et al.* 2023, Zhang and Zhao 2023). Consequently, enrichment with GTEx eQTLs likely represents only a subset of the regulatory variants captured by our cell type-resolved analysis.

In the future, as larger and higher-resolution datasets become available, models could be trained at the single-cell level to fully leverage expression variability between individual cells. More sophisticated machine learning methods, such as neural networks, which currently face challenges for gene-specific single-cell models due to their large number of hyperparameters, could be applied. However, these models are considerably more computationally intensive, which is an important consideration given that tens of thousands of models need to be trained. With MetaFR, we provide a highly efficient pipeline for accurate gene expression prediction, including an optional aggregation of individual cells into meta-cells to enhance signal, making it well-suited for application to large-scale datasets in future studies.

## Methods

### Data pre-processing

We used the multiome 10× Genomics PBMC dataset ^1^. We downloaded the filtered feature barcode matrix, the ATAC per fragment information, and index files. We applied the filtering steps from the Seurat weighted-nearest-neighbor analysis vignette (https://satijalab.org/seurat/articles/weighted_nearest_neighbor_analysis): all cells with scATAC counts >70 000 and <5 000, and with scRNA counts <1 000 and >25 000 were filtered. In addition, cells with more than 20% mitochondrial reads were excluded, resulting in 10 412 high quality cells. The gene expression was CPM normalized (scaling factor 10 000). ATAC and RNA cell integration, was done using the Seurat vignette for that purpose (https://satijalab.org/seurat/articles/seurat5_atacseq_integration_vignette). The main steps included computing gene activity scores (GeneActivity() function), using them to identify anchors (mutual nearest neighbors via FindTransferAnchors()) for CCA-based integration, and finally co-embedding RNA and ATAC cells with TransferData(). Seurat (Hao *et al.* 2024) v4.3.0 was used.

### MetaFR pipeline

The subsequent steps have been fully automated with Nextflow (version 25.04.6). The required input files for our pipeline are a fragment file containing the fragment positions, barcodes and the total read count and the index file for the epigenetic modality, and the gene expression count matrix. In addition, a Seurat object containing the integrated RNA and ATAC cells is needed when applying the meta-cell aggregation step.

#### Meta-cell creation

For generating the optional meta-cells, a Seurat object containing the integrated RNA and ATAC cells is required as input. Here, single-cell RNA-seq and ATAC-seq data can be either measured in the same cell (multiome) or different cells (unpaired). Two main steps were performed for signal summarization: first, the cells containing the RNA-seq signal were clustered using a k-medoid approach based on the similarity of their gene activity profiles. The cluster centers represent the RNA meta-cells. The clustering was performed separately within each annotated cell type. The cell type annotation was obtained from Stuart *et al.* (2019). Next, the cells containing the epigenetic signal were assigned to the closest RNA meta-cell with a k-nearest-neighbor (kNN) approach. For both steps, the Euclidean distance was applied as the distance metric. For our analyses, the parameter *k*, corresponding to the number of cells per meta-cell, was varied between 50, 100 and 150. Varying this parameter impacts the total number of meta-cells. Our implementation is available as an R package **MetaCellaR**: https://github.com/fba67/MetaCellaR.

#### Meta-cell filtering

For all analyses, we filtered meta-cells with less than 200 000 ATAC-seq counts.

#### Activity Binning

Prior to model learning, the feature matrices were generated for each gene. They contain the ATAC signal quantified within equal-sized bins $b_{1}\ldots b_{m}$ in a 1 MB window centered on the most $5^{\prime }$ GENCODE (The GENCODE consortium 2019) TSS of a gene for each sample $s_{1}\ldots s_{n}$ (single-cell or meta-cell). We varied the bin sizes between 100, 250 and 500 basepairs. The ATAC activity was TF-IDF normalized and for activity binning, the Signac (Stuart *et al.* 2021) FeatureMatrix function was used.

#### Random forest regression

For the model training we applied a Random Forest regression approach on the binned epigenetic data with the RandomForestRegressor function from the scikit-learn library in Python. The response was min-max scaled to the range [0,1]. The default parameter settings were used for all analyses:

n_estimators=100, criterion=’squared_error’,max_depth=None,

min_samples_leaf=1, min_weight_fraction_leaf=0.0,

max_features=1.0, max_leaf_nodes=None, min_samples_split=2,

min_impurity_decrease=0.0, bootstrap=True, monotonic_cst=None,

oob_score=False, n_jobs=None, random_state=0, verbose=0,

warm_start=False, ccp_alpha=0.0, max_samples=None

### Performance assessment

For the single-cell and meta-cell setups we randomly held-out 20% of the cells or meta-cells for model testing. All 19 cell types are present in both, train and test set for the single-cell setup. For the meta-cell setups, 18 cell types are present in the training set, CD4 Naive meta-cells are only present in the test set. To evaluate prediction accuracy, the Spearman correlation, as well as the Mean-Squared-Error (MSE) was calculated between predicted and actual gene expression for each setup on the corresponding test set.

### eQTL enrichment analysis

To evaluate differences in meta-cell and single-cell setups in their ability to capture interactions supported by eQTL gene-pairs on the PBMC dataset, the *Whole Blood* tissue from GTEx was chosen. The fine-mapping method with the highest number of eQTL-gene pairs, DAP-G, was selected (‘GTEx_v8_finemapping_DAPG.CS95.txt.gz’ file). The 1 000 genes with the highest variability in the observed expression were considered for this analysis. For each MetaFR setup (50, 100 and 150 cells per meta-cell and single-cell) we calculated approximated SHAPley values using the TreeSHAP (Lundberg *et al.* 2020) algorithm (shap library version 0.47.1) to obtain the regions that are important for a model’s prediction. To compare single-cell and meta-cell setups, the mean SHAP value for each cell type was calculated. To rank the genomic bin-gene interactions across genes, the absolute SHAP values were scaled to interquantile range (IQR) using the RobustScaler function from the scikit-learn library in Python. In addition, the IQR-scaled SHAP values $\mathrm{sha}p_{g,b}$ for each interaction g,b between gene g and bin b were weighted with a distance-decay function (Chen and Keleş 2024):


(1) $$\mathrm{adjus}t_{g,b}=(\mathrm{sha}p_{g,b}+\epsilon )\cdot e^{\frac{\mathrm{dis}t_{g,b}}{d_{0}}},$$

where ϵ was set to 0.05, $\mathrm{dis}t_{g,b}$ represents the distance between a gene’s most $5^{\prime }$ TSS and genomic bin, and weight decay scaling parameter $d_{0}=200\;000$bp. Then, the normalized enrichment score of eQTLs in the top 100 000 highest ranked interactions was calculated using the GSEApy (Fang *et al.* 2023) pre-rank function (v.1.1.2) in a cell type-specific manner. The number of permutations was set to 100. The weighting parameter p was set to zero. In order to ensure the same number of interactions supported by eQTL-gene pairs for each cell type, the number of overlapping eQTL-gene pairs was reduced to the minimum overlap across all cell types.

### Performance comparison MetaFR and SCARlink

SCARlink (Mitra *et al.* 2024) employs a 250 kb window around the gene body for each gene. The size of the window surrounding the most $5^{\prime }$ GENCODE TSS of a gene was set to 1 MB for MetaFR. The epigenetic signal was quantified in 500 bp bins for both methods. The gene expression was CPM normalized with scaling factor 10 000. Following the default practice for SCARlink, the input gene set was defined as the 2000 most variable genes. For MetaFR, all 36 601 genes present in the dataset without any filtering on the expression variance were used for model training. For SCARlink, the ArchR (Granja *et al.* 2021) AddTileMatrix function was used for generating the feature matrices. All analyses were performed on single-cell level on the same set of test cells. 20% of the data set was randomly selected for testing. For MetaFR, predicted and observed expression values were multiplied by a constant factor reflecting SCARlink’s internal normalization ($10^{6}/{\text{nCount}}_{\text{RNA}}$), ensuring that MSE was computed on a comparable scale.

## Supplementary Material

btag299_Supplementary_Data

## Data Availability

We downloaded the PBMC dataset from the 10× Genomics Website: https://www.10xgenomics.com/datasets/10-k-human-pbm-cs-multiome-v-1-0-chromium-x-1-standard-2-0-0. The pipeline and manuscript code are publicly available on GitHub: https://github.com/SchulzLab/MetaFR and archived on Zenodo at DOI: 10.5281/zenodo.19501506.

## Footnotes

1
https://www.10xgenomics.com/datasets/10-k-human-pbm-cs-multiome-v-1-0-chromium-x-1-standard-2-0-0
