# Supplementary material for: Predicting gene-specific regulation with transcriptomic and epigenetic single-cell data
Source: Bioinformatics. 2026 May 15;42(6):btag299. doi: 10.1093/bioinformatics/btag299 (PMC13283429; doi:10.1093/bioinformatics/btag299)
Supplement: btag299_Supplementary_Data [file btag299_supplementary_data.zip › MetaFR_Supplementary_Material.pdf]

Supplemental Material for the paper:

Predicting gene-specific regulation with transcriptomic and epigenetic single-cell data

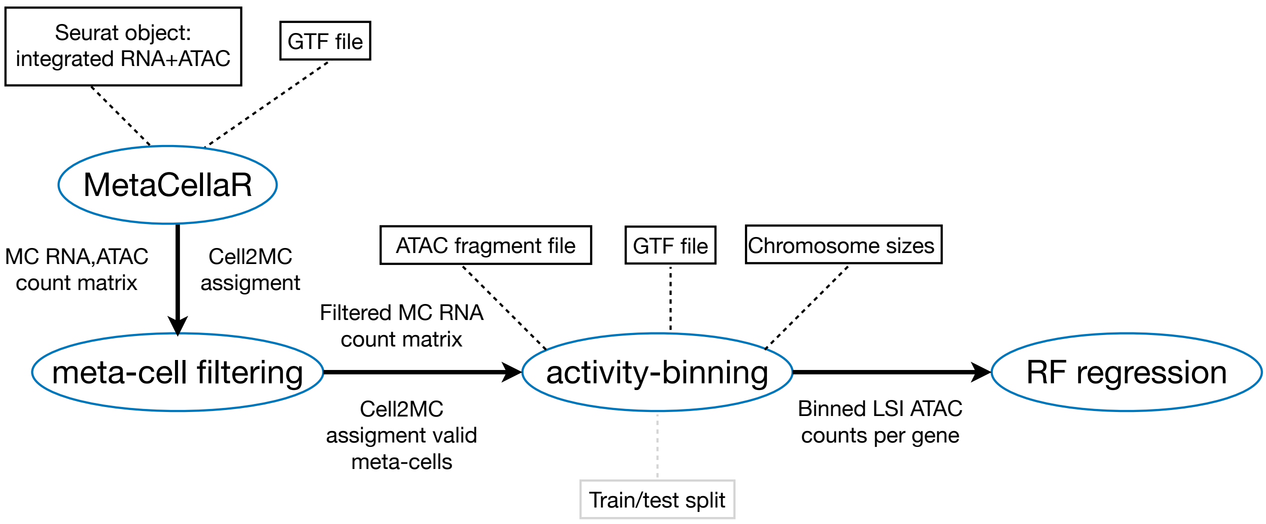

Fig. 1. Flowchart MetaFR Nextflow pipeline. MetaCell creation (MetaCellaR and meta-cell filtering) is optional. For MetaCellaR a Seurat object containing the integrated RNA and ATAC cells is required as input together with a GTF file. The output is the gene expression and ATAC count matrix on meta-cell level, as well as the assignment of cells to meta-cells. In the meta-cell filtering step, meta-cells that contain less than 200, 000 ATAC reads are excluded. This threshold can be adjusted by the user. For the activity-binning step, additional input files are needed: the ATAC fragment file and chromosome sizes. Optionally, a train-test partition of the cells/meta-cells can be provided. If not, a random train-test split (default = 20%) will be applied. The LSI-normalized ATAC count matrices are then used to train a RF regression model per gene.

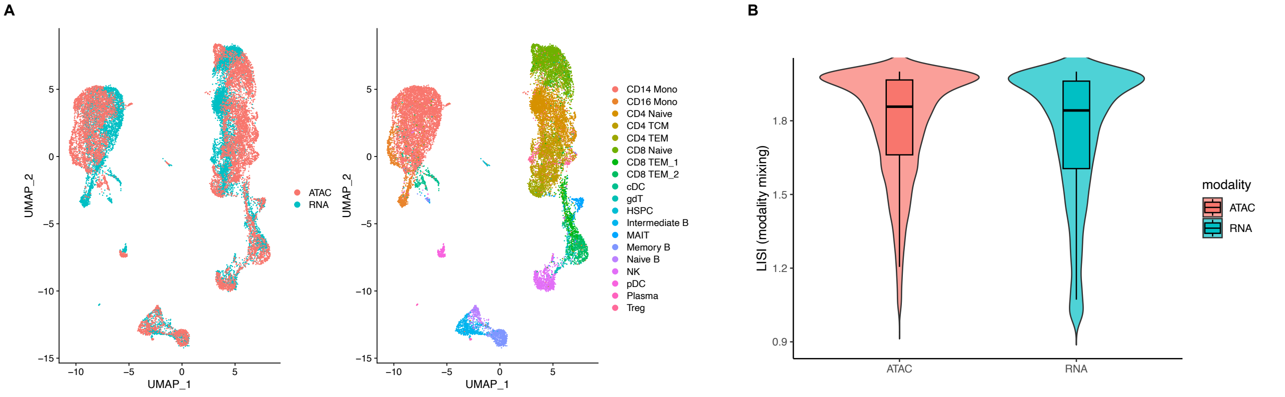

Fig. 2. Assessment Seurat Integration multiome 10X PBMC dataset. **A** UMAP of the 10X PBMC dataset (https://www.10xgenomics.com/datasets/10-k-human-pbm-cs-multiome-v-1-0-chromium-x-1-standard-2-0-0) showing the integrated RNA and ATAC cells generated using Seurat (Stuart et al. [2019]). The same embedding colored by cell type annotation from Stuart et al. [2019]. (B) Local Inverse Simpson's Index (LISI) (Korsunsky et al. [2019]) for ATAC and RNA cells.

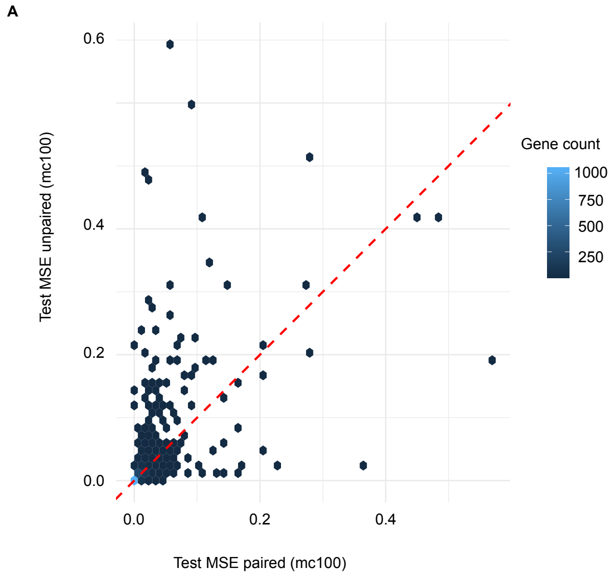

Fig. 3. Model performance assessment for paired and unpaired meta-cell generation. A Test error (MSE) between predicted and actual gene expression of models trained with paired (x-axis) or unpaired (y-axis) meta-cell aggregation for 100 cells per meta-cell. To compare our approach to a paired strategy that leverages pairing information in multiome single-cell data, we skipped the unsupervised ATAC-to-RNA assignment and instead directly assigned corresponding ATAC cell barcodes to the RNA meta-cells. We evaluated this implementation on a subset of approximately 2,000 genes across five randomly selected chromosomes on the same set of test meta-cells.

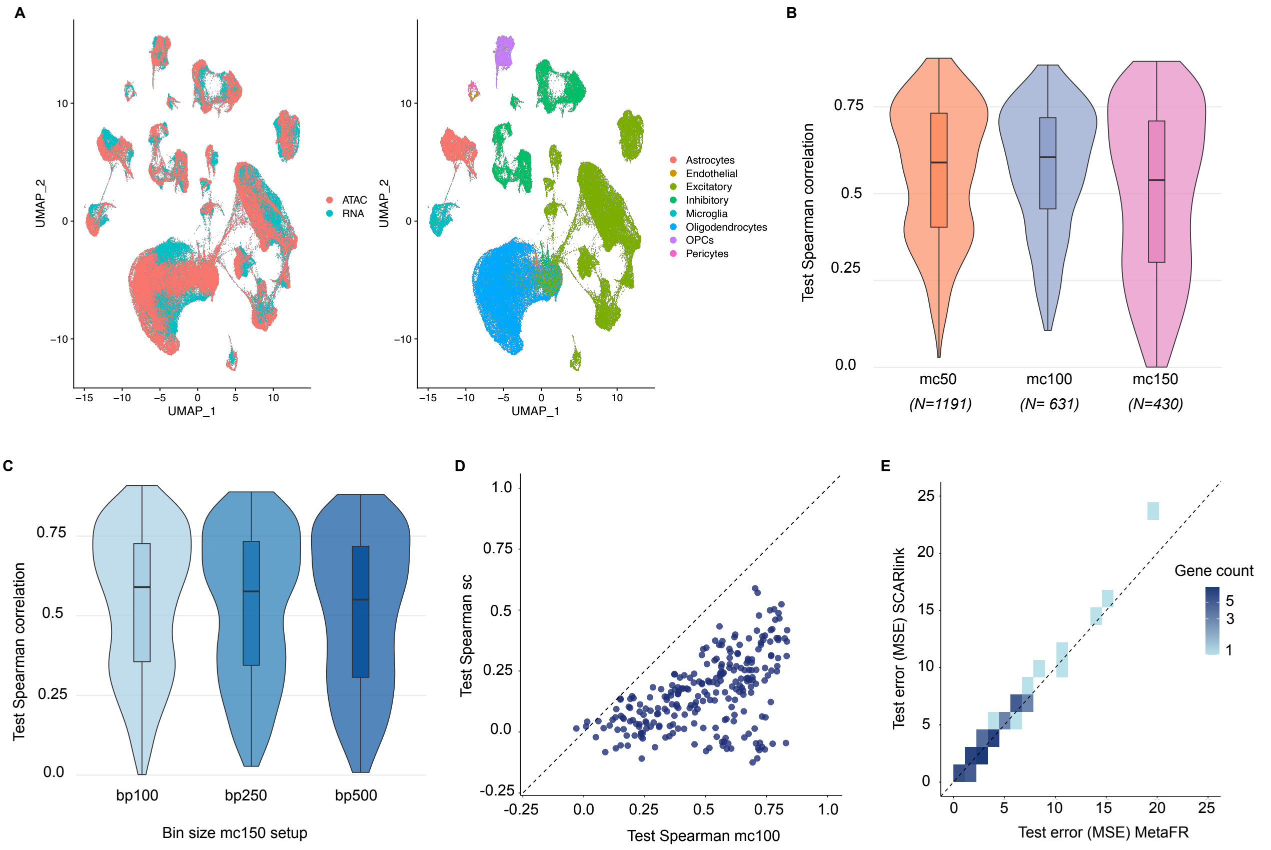

Fig. 4. Performance assessment on multiome Brain dataset. Models were trained for the 500 most variable genes identified using Seurat's VariableFeatures function (Stuart et al. [2019]). To assess model accuracy Spearman correlation and Mean-Squared-Error (MSE) were calculated between predicted and actual gene expression on a test set (20% of all data). **A** *(Left)* UMAP of the brain dataset Anderson et al. [2023] showing the integrated RNA and ATAC cells generated using Seurat. *(Right)* The same embedding colored by cell type annotation from Anderson et al. [2023]. The dataset contains $105,332$ nuclei isolated from cortical tissues of 7 Alzheimer's disease (AD) and 8 unaffected donors. **(B+C)** Center line is median, boxlimits correspond to IQR, whiskers to $1.5x$ IQR. Outliers are not shown. A paired Wilcoxon signed-rank test was performed between gene sets of different setups. **B** Correlation on test data for the meta-cell setups with 50, 100 and 150 cells aggregated into one meta-cell for 333 genes, where all setups could obtain a model with test correlation > 0. **C** Correlation on test data for the meta-cell setup with 150 cells per meta-cell and bin sizes varying between 100, 250 and 500 base-pairs. **D** Test correlation for meta-cell setup with 100 cells per meta-cell and aggregated single-cell predictions. Aggregated single-cell predictions are the summed predictions for cells that belong to the same meta-cell. **E** Test error of MetaFR and SCARlink for 125 genes for which both methods produced a model. 8 outliers (MSE $>$ 25) were excluded for visualization purposes, of which 7 showed a higher MSE for SCARlink. Runtime per gene for SCARlink was approximately 20.82 minutes, for MetaFR approximately 1.47 minutes on an Intel Xeon CPU node with 64 Cores at $2.6 - 3.3$ GHz Frequency and 384 GiB RAM using 25 cores.

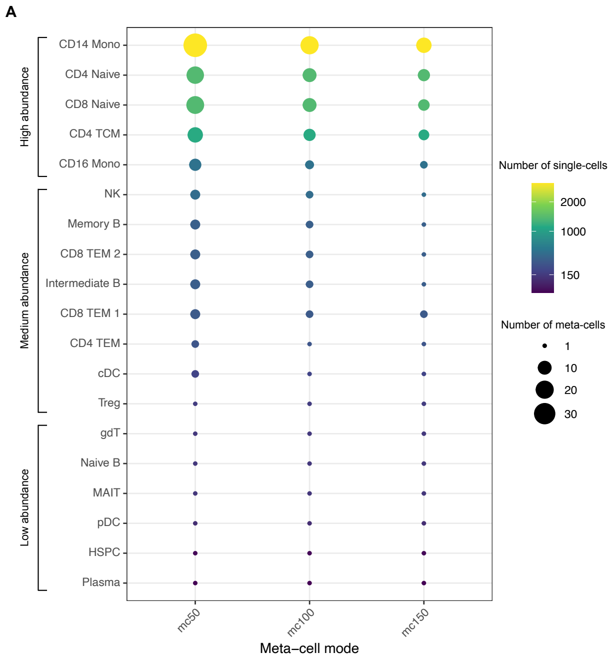

Fig. 5. Assessment meta-cell resolution across cell types. A Dot plot showing the number of meta-cells generated for each cell type under different meta-cell aggregation settings. Dot size indicates the number of meta-cells, while color represents the number of single cells per cell type. Cell types are grouped by abundance: low (< 150 cells), medium (< 500 cells), and high (> 500 cells).

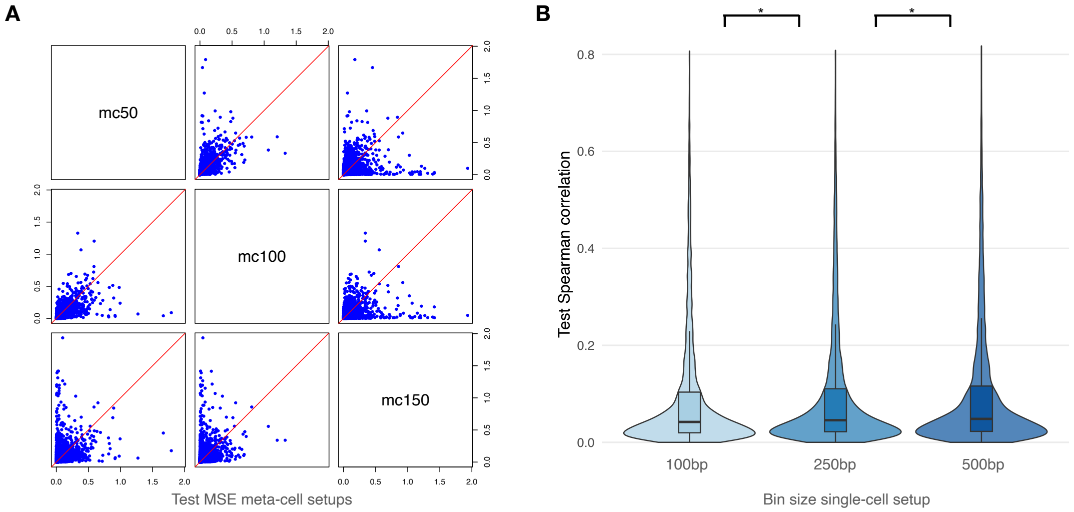

Fig. 6. Model performance assessment for meta-cell (mc) and single-cell (sc) MetaFR setups. To assess model accuracy the Spearman correlation was calculated between predicted and actual gene expression on a test set (20% of all data). **A** MSE on test data for the meta-cell setups with 50, 100 and 150 cells aggregated into one meta-cell for 19,306 genes. The MSE was calculated on the log-transformed CPM expression values. **B** Correlation on test data for the single-cell setup and bin sizes of 100, 250 and 500 base-pairs. Center line is median, boxlimits correspond to IQR, whiskers to 1.5x IQR. Outliers are not shown. A paired Wilcoxon signed-rank test was performed between gene sets of different setups (p-value ≤ 0.05 indicated with an asterisk).

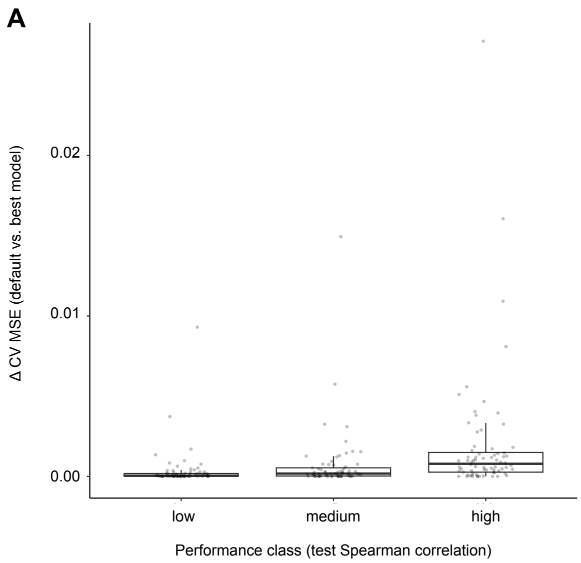

Fig. 7. Robustness of Random Forest performance to hyperparameter selection. A Difference in Cross-Validation Mean-Squared-Error (ΔCV-MSE) between the default parameter setting and the best performing setting for 216 genes. Model performance was evaluated using 3-fold cross-validation, and ΔCV-MSE was defined as the difference between the mean cross-validation error of the default and best-performing parameter setting. Genes were grouped according to their test correlation (Spearman) between predicted and actual expression: < 0.3 (low), < 0.6 (medium) and > 0.6 (high). Models were trained on meta-cells containing 100 cells per meta-cell.

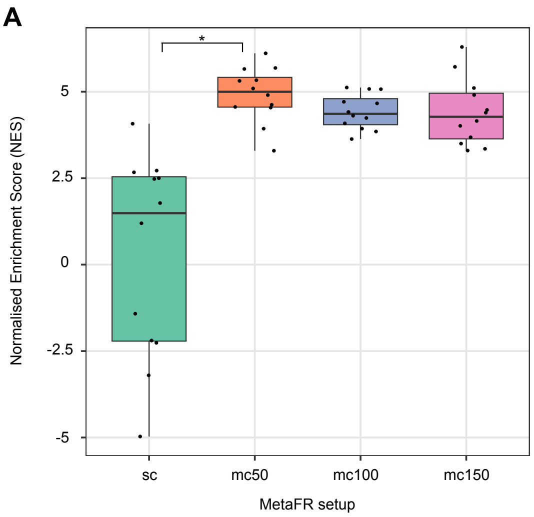

Fig. 8. Comparison between single-cell (sc) and meta-cell (mc) MetaFR setups without distance weighting. A Cell type-specific GSEA Normalized Enrichment Score (NES) (Fang et al. [2023]) of GTEx (The GTEx Consortium [2020]) whole blood eQTLs in the top 100,000 ranked interactions of the 1,000 most variable genes. Interactions were ranked across genes using IQR-scaled absolute SHAP values. Cell types with fewer than 100 cells were excluded. The Center line denotes the median, boxlimits correspond to IQR, whiskers to 1.5x IQR. A paired Wilcoxon signed-rank test was performed between gene sets of different setups. P-value 0.05 indicated with an asterisk.

Fig. 9. Investigation of gene characteristics that impact model performance. Assessed gene characteristics are gene length, gene density ($=$ number of genes within the 1 MB window around the gene), number of annotated GENCODE transcripts, gene expression sparsity (fraction of zeros in expression across test cells), number of annotated GENCODE TSSs and total exon length. **A** The best-performing gene sets for the aggregated single-cell sc (4,615 genes) and meta-cell mc100 setup with 100 cells per meta-cell (7,172 genes) are defined as the models that achieve a minimum performance of 0.3 test correlation and where one method outperforms the other by > 0.1. **B+C** Gene sets were classified based on their test correlation for single-cell (not aggregated) and meta-cell (100 cells per mc): < 0.1 is defined as failed, < 0.5 as medium and > 0.5 as high. **B** MetaFR setup with 100 cells per meta-cell with $7,106$ high performing, $5,536$ medium performing and $6,111$ failed genes. **c** Single-cell MetaFR with 159 high performing, 1970 medium performing and $16,319$ failed genes. Center line is median, box limits correspond to IQR, whiskers to $1.5\times$ IQR. An unpaired Mann–Whitney U test was performed between the best-performing gene sets. P-value ≤ 0.05 indicated with an asterisk.

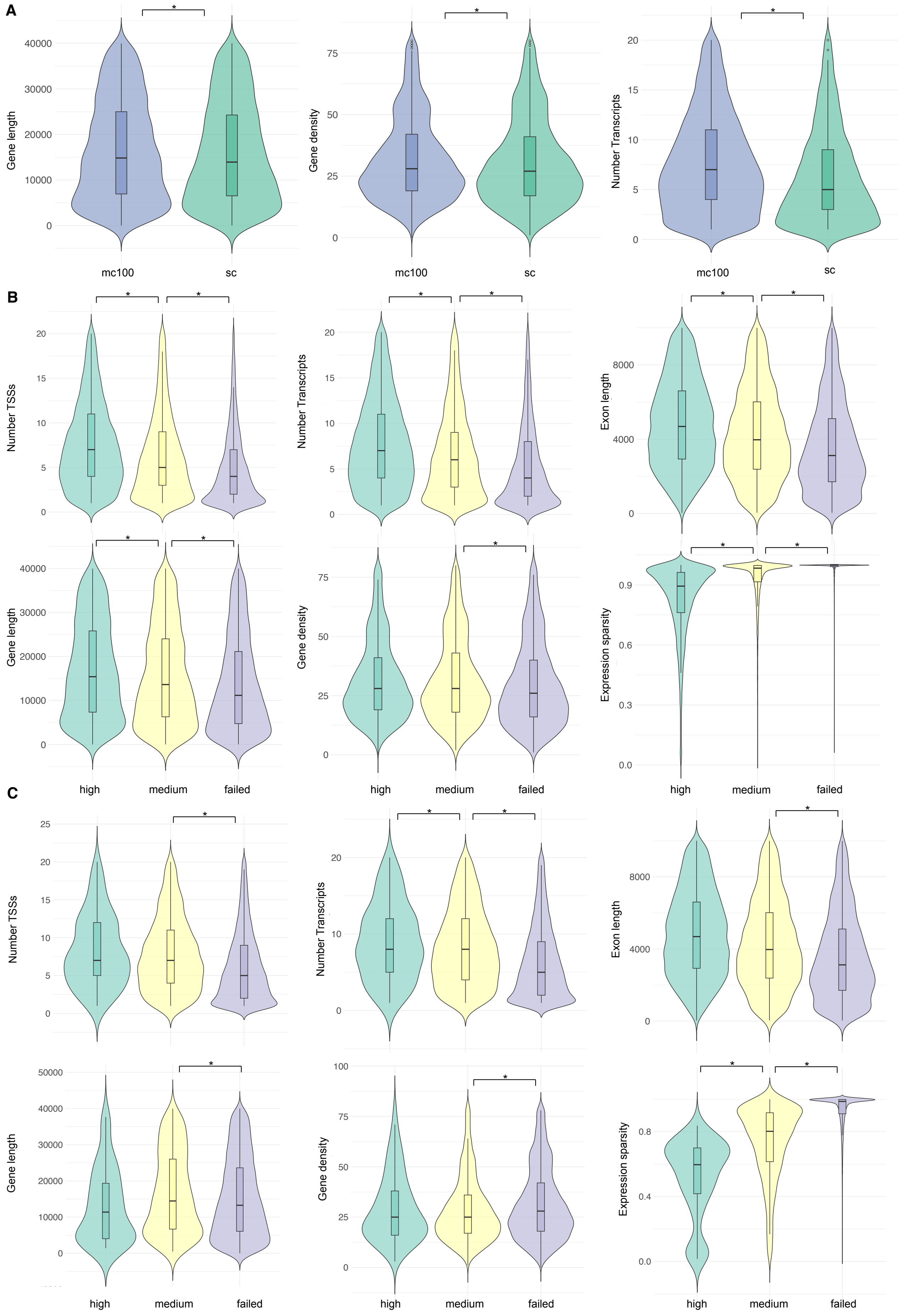
A
Gene length
Gene density
Number Transcripts
mc100
sc
B
Number TSSs
Number Transcripts
Exon length
Gene length
Gene density
Expression sparsity
high
medium
failed
C
Number TSSs
Number Transcripts
Exon length
Gene length
Gene density
Expression sparsity
high
medium
failed

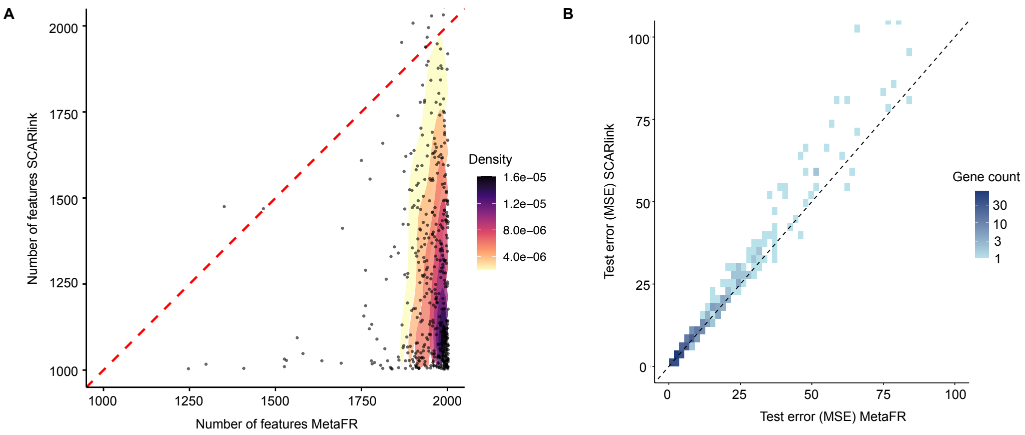

Fig. 10. Comparison to state-of-the-art method SCARlink on feature subset. A Number of features used by MetaFR and SCARlink for 495 genes, for which both methods produced a model. **B** Test error (MSE) between predicted and actual expression after reducing MetaFR features to 1, 234 (median SCARlink feature count) by symmetrically trimming the most distal regions around the TSS.

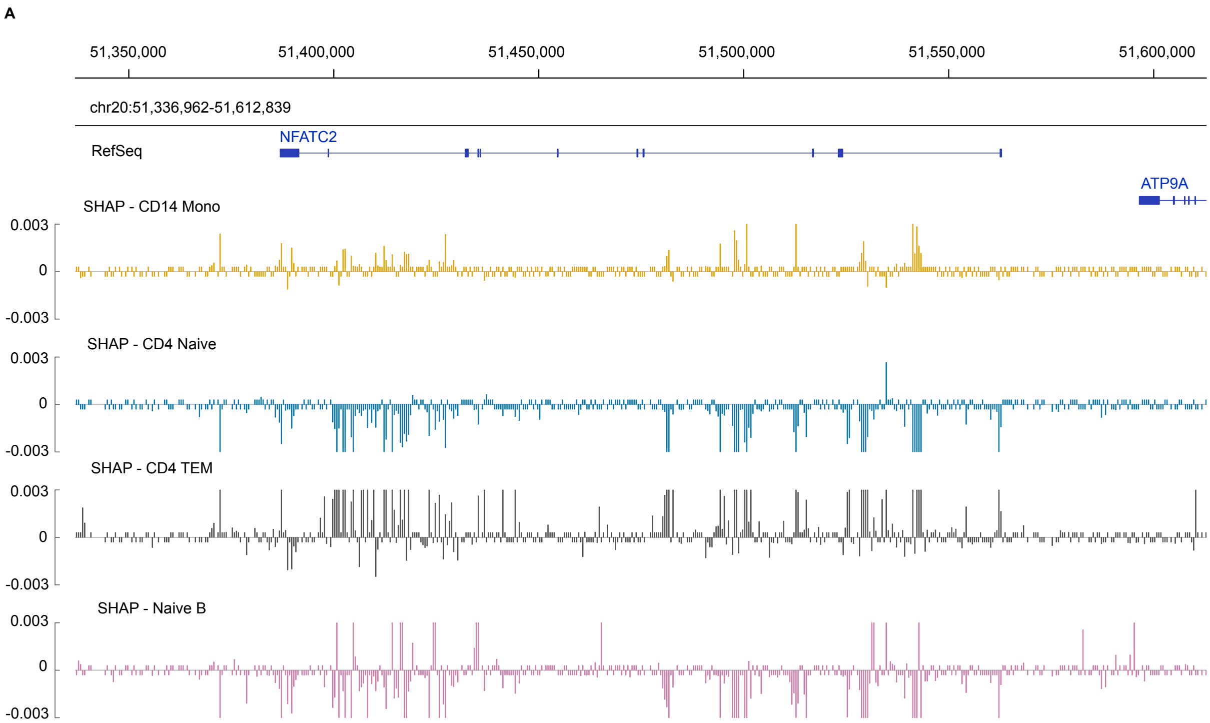

Fig. 11. SHAP importance profiles at the NFATC2 locus across cell types. A Integrative Genomics Viewer (IGV) visualization (Robinson et al. [2011], Shannon et al. [2025]) showing SHAP importance scores across selected cell types in the 10x PBMC dataset.

Supplementary Methods

Hyperparameter assessment

Random Forest hyperparameters were explored using `RandomizedSearchCV` from the scikit-learn library in Python. The following hyperparameter ranges were considered:

- `n_estimators` = [100, 200, 400]
- `max_depth` = [None, 3, 5, 8]
- `min_samples_split` = [2, 5, 10]
- `min_samples_leaf` = [1, 2, 5]
- `max_features` = [”sqrt”, 0.5, 1.0]

From these ranges, 19 hyperparameter configurations were randomly sampled (`n_iter=19`) and model performance was evaluated using a 3-fold cross-validation (`cv=3`). Mean-Squared-Error (MSE) between predicted and actual gene expression was used as the scoring metric. In addition, the default parameter setting was evaluated separately using the same cross-validation procedure. To assess robustness of model performance to hyperparameter selection, we compared the difference between CV-error for each parameter setting and the best-performing parameter setting for a subset of 216 genes. These genes were selected based on the Spearman correlation between predicted and actual expression in the held-out test data set, spanning 3 performance classes: low (< 0.3), medium (< 0.6) and high (> 0.6). The models were trained on meta-cells containing 100 cells per meta-cell.

Cell type-specific TF enrichment analysis

A gene set of cell type-specific marker genes present in the multiome 10x PBMC dataset was defined based on differential expression using Seurat’s FindMarkers function (log fold change > 0.25), resulting in approximately $2,000$ genes. For each cell type, region-gene interactions were ranked across genes using z-score normalized SHAP values, and the top $1,000$ interactions were selected. Only cell types with more than one meta-cell were included. The models used in this analysis were trained on 100 cells per meta-cell and 500 bp bins. To assess whether top-ranked interactions are enriched for cell type-specific regulatory programs, transcription factor binding affinities to genomic regions were estimated using TRAP (Roider et al. [2007]). Based on these affinities, PASTAA (Roider et al. [2009]) was applied to identify TFs whose predicted binding is enriched in the top-ranked interactions, followed by false discovery rate (FDR) correction using the Benjamini–Hochberg procedure (Benjamini and Hochberg [1995]).

Supplementary Files

The following supplementary files are provided:

- **All GO terms for MetaFR with 100 cells per meta-cell** `go_enrichment_mc100_spearman_q0_500bp_all_terms.tsv` — full list of 8,804 significant Gene Ontology terms.
- **All GO terms for single-cell MetaFR** `go_enrichment_aggr_sc_spearman_q0_500bp_all_terms.tsv` — full list of 15 significant Gene Ontology terms.
- **All cell type-specific TF enrichments for MetaFR with 100 cells per meta-cell** `pastaa_results_FDR_pbmc.tsv` — full list of FDR-corrected transcription factor enrichment results (only cell types with more than one meta-cell included).

References

A. G. Anderson, B. B. Rogers, J. M. Loupe, I. Rodriguez-Nunez, S. C. Roberts, L. M. White, J. N. Brazell, W. E. Bunney, B. G. Bunney, S. J. Watson, J. N. Cochran, R. M. Myers, and L. F. Rizzardi. Single nucleus multiomics identifies zeb1 and mafb as candidate regulators of alzheimer's disease-specific cis-regulatory elements. *Cell Genomics*, 3(3):100263, Mar. 2023. ISSN 2666-979X. doi: 10.1016/j.xgen.2023.100263.

Y. Benjamini and Y. Hochberg. Controlling the False Discovery Rate: A Practical and Powerful Approach to Multiple Testing. *Journal of the Royal Statistical Society. Series B (Methodological)*, 57(1):289–300, 1995. URL `http://www.jstor.org/stable/2346101`.

Z. Fang, X. Liu, and G. Peltz. GSEApy: a comprehensive package for performing gene set enrichment analysis in Python. *Bioinformatics*, 39(1):btac757, Jan. 2023. ISSN 1367-4811. doi: 10.1093/bioinformatics/btac757. URL `https://academic.oup.com/bioinformatics/article/doi/10.1093/bioinformatics/btac757/6847088`.

I. Korsunsky, N. Millard, J. Fan, K. Slowikowski, F. Zhang, K. Wei, Y. Baglaenko, M. Brenner, P.-r. Loh, and S. Raychaudhuri. Fast, sensitive and accurate integration of single-cell data with harmony. *Nature Methods*, 16(12): 1289–1296, Dec. 2019. ISSN 1548-7105. doi: 10.1038/s41592-019-0619-0.

J. T. Robinson, H. Thorvaldsdóttir, W. Winckler, M. Guttman, E. S. Lander, G. Getz, and J. P. Mesirov. Integrative genomics viewer. *Nature biotechnology*, 29(1):24–26, Jan. 2011. ISSN 1087-0156. doi: 10.1038/nbt.1754.

H. G. Roider, A. Kanhere, T. Manke, and M. Vingron. Predicting transcription factor affinities to dna from a biophysical model. *Bioinformatics*, 23(2):134–141, Jan. 2007. ISSN 1367-4811. doi: 10.1093/bioinformatics/btl565.

H. G. Roider, T. Manke, S. O'Keeffe, M. Vingron, and S. A. Haas. Pastaa: identifying transcription factors associated with sets of co-regulated genes. *Bioinformatics*, 25(4): 435–442, Feb. 2009. ISSN 1367-4803. doi: 10.1093/bioinformatics/btn627.

P. Shannon, A. Gladki, and K. Scigocka. *igvShiny: igvShiny: a wrapper of Integrative Genomics Viewer (IGV - an interactive tool for visualization and exploration integrated genomic data)*, 2025. URL `https://github.com/gladkia/igvShiny`. R package version 1.5.2.

T. Stuart, A. Butler, P. Hoffman, C. Hafemeister, E. Papalexi, W. M. Mauck, Y. Hao, M. Stoeckius, P. Smibert, and R. Satija. Comprehensive integration of single-cell data. *Cell*, 177(7):1888–1902.e21, 2019. ISSN 0092-8674. doi: https://doi.org/10.1016/j.cell.2019.05.031.

The GTEx Consortium. The GTEx consortium atlas of genetic regulatory effects across human tissues. *Science*, 369(6509): 1318–1330, 2020. doi: 10.1126/science.aaz1776.
